# Perception of Emotional Facial Expressions in Amyotrophic Lateral Sclerosis (ALS) at Behavioural and Brain Metabolic Level

**DOI:** 10.1371/journal.pone.0164655

**Published:** 2016-10-14

**Authors:** Helena E. A. Aho-Özhan, Jürgen Keller, Johanna Heimrath, Ingo Uttner, Jan Kassubek, Niels Birbaumer, Albert C. Ludolph, Dorothée Lulé

**Affiliations:** 1 Department of Neurology, University of Ulm, Ulm, Germany; 2 Institute of Medical Psychology and Behavioral Neurobiology, Eberhard-Karls-University of Tübingen, Tübingen, Germany; 3 The Wyss Center for Bio and Neuroengineering, Geneva, Switzerland; Universitat Regensburg, GERMANY

## Abstract

**Introduction:**

Amyotrophic lateral sclerosis (ALS) primarily impairs motor abilities but also affects cognition and emotional processing. We hypothesise that subjective ratings of emotional stimuli depicting social interactions and facial expressions is changed in ALS. It was found that recognition of negative emotions and ability to mentalize other’s intentions is reduced.

**Methods:**

Processing of emotions in faces was investigated. A behavioural test of Ekman faces expressing six basic emotions was presented to 30 ALS patients and 29 age-, gender and education matched healthy controls. Additionally, a subgroup of 15 ALS patients that were able to lie supine in the scanner and 14 matched healthy controls viewed the Ekman faces during functional magnetic resonance imaging (fMRI). Affective state and a number of daily social contacts were measured.

**Results:**

ALS patients recognized disgust and fear less accurately than healthy controls. In fMRI, reduced brain activity was seen in areas involved in processing of negative emotions replicating our previous results. During processing of sad faces, increased brain activity was seen in areas associated with social emotions in right inferior frontal gyrus and reduced activity in hippocampus bilaterally. No differences in brain activity were seen for any of the other emotional expressions. Inferior frontal gyrus activity for sad faces was associated with increased amount of social contacts of ALS patients.

**Conclusion:**

ALS patients showed decreased brain and behavioural responses in processing of disgust and fear and an altered brain response pattern for sadness. The negative consequences of neurodegenerative processes in the course of ALS might be counteracted by positive emotional activity and positive social interactions.

## Introduction

Amyotrophic lateral sclerosis (ALS) is a multi-system disorder with the most prominent feature of progressive pyramidal tract pathology but also involving extra-motor cortical areas and other spinal systems [1]. Prefrontal cortical dysfunctions may occur in 30–40% of ALS patients [2]. Furthermore, ALS patients may present with reduced memory capacity for i.e. emotional material [3]. Other domains of emotional processing are similarly affected such as evaluation of emotional stimuli of social situations. ALS patients regard negative pictures as less arousing and more positive [4]. Neurodegeneration of cortical [1,5] and limbic structures such as the amygdala [6] and nucleus accumbens [7] might affect emotional processing abilities especially for aversive emotional information [8] but environmental factors may also contribute to these changes [9]. Furthermore, reduced afferent peripheral inflow (i.e. “somatic markers”) [10] to subcortical and cortical networks such as the limbic system may explain variance in emotional processing and “dampening” of negative feelings.

As ALS patients often face an increasing dependency on others, changes in emotional perception might become especially burdensome for the caretakers and clinical staff [11]. Evidence from previous studies suggests cortical compensatory functional reorganization especially in the early course of ALS [12]. Whether these reorganisation processes have a compensatory effect at behavioural level is not clear.

In the current study, emotional processing of facial cues was measured in ALS patients compared to healthy participants. Using functional magnetic resonance imaging (fMRI), we explored brain processing of emotional facial expressions in a subgroup of ALS patients and healthy participants.

Furthermore, degree of brain activity was correlated with degree of depression and number of social contacts in everyday life across all the subjects, as we have hypothesized that positive contact with caregivers and family reduces negative emotional perception and improves positive emotional response in ALS [9].

## Methods

### Participants

Thirty patients (16 females; 21 with spinal, 9 with bulbar onset; mean age 60±10 years) diagnosed with probable or definite ALS according to the revised El Escorial criteria [13] by a board certified neurologist, participated in the emotion recognition task and a subgroup of fifteen patients (5 females, all sporadic cases, all spinal onset, mean age 54±12 years) in the fMRI paradigm. ALS patients were consecutively recruited from the outpatient clinic of the Department of Neurology at the University of Ulm. All the patients had at least six months between the diagnosis and testing.

Patients’ disease status was assessed with ALS functional rating scale revised (ALS-FRS-R) [14]. Patients who met the criteria of frontotemporal dementia (FTD) were excluded. N = 12 patients were intermittently treated by non-invasive ventilation and had shortness of breath when lying supine in the scanner and N = 4 patients used a wheelchair. They were all excluded from the fMRI study.

Twenty-nine age, gender and education matched healthy volunteers (8 females, mean age 61±8 years) served as controls for the emotion recognition task and fourteen of them (7 females, mean age 61±10 years) for the fMRI paradigm. Healthy controls were contacted via email by the organizing committee for senior education at the University of Ulm. All the patients and healthy controls were also included in another study reported previously [15].

The participants were all right-handed [16] with normal vision. None of the patients and healthy controls had a history of neurological or psychiatric disorder. N = 24 patients received Riluzole, including all the patients participating in the fMRI paradigm. The participants received no other medication affecting central nervous system.

The study was approved by the Ethics Committees of the Universities of Ulm and Tübingen (174/2008) and was performed in accordance with the ethical standards laid down in the 1964 Declaration of Helsinki. All healthy controls and patients gave written informed consent prior to inclusion in the study.

### Neuropsychological assessment

To make sure the participants were able to understand instructions they all were screened for major cognitive deficits by assessing Mini Mental State Examination (MMSE) [17]. Patients with cognitive deficits (cut-off ≤ 26) were excluded. In addition, all the participants were assessed with the Beck`s Depression Inventory (BDI, range for mild depression 12–19) [18].

Furthermore, the participants of the fMRI paradigm received more extensive neuropsychological assessment. They were screened for cognitive deficits by assessing the MMSE and a Mehrfachwahl-Wortschatz-Intelligenztest (MWT-B) measuring premorbid crystallized verbal intelligence [19]. Additionally, specific neuropsychological testing was focused on frontal lobe functions including verbal fluency (Regensburg Fluency Test) [20], design fluency (5-Point Fluency Test) [21] and attention (Symbol Digit Modalities Test) [22]. Social contacts were assessed by using an in-house questionnaire determining social activity as the number of people seen (at least one minute per person) on average per day and the average time spent with each person.

### Experimental design

#### Facial emotion processing at behavioural level

ALS patients and healthy controls first performed a verbal rating of Ekman faces stimuli (facial expressions of emotion test; FEEST) [23]. For the verbal rating task participants viewed 60 black-and-white pictures of facial expressions of basic emotions (10 stimuli of each emotion: anger, disgust, fear, sadness, surprise and happiness) on a computer screen. Participants were asked to choose either of six displayed emotions according to what he/she most likely saw expressed on the face. Faces were displayed for 6s but participants had unlimited time to tick the box of any of the six displayed emotions.

#### Facial emotion processing at functional cortical level (fMRI)

**Fig 1:**
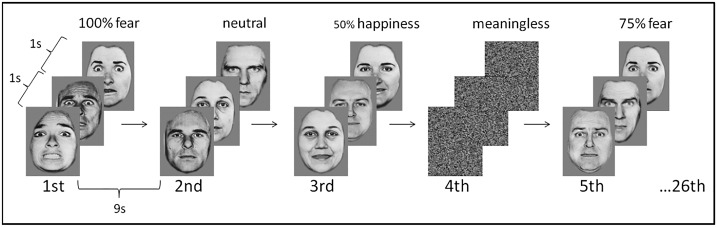
Example sequence of the event related fMRI paradigm of different intensities of emotions and meaningless and neutral stimuli. In total three trials, each including 26 stimulation sequences was presented. A stimulation sequence consisted of a block of three stimuli of one second interleaved by rest epochs of one second. Each block was followed by a rest period of nine seconds. Stimulus blocks of different emotions (anger, disgust, fear, sadness, surprise and happiness; here only fear and happiness are displayed as examples) with different intensities and neutral and meaningless stimuli were presented in a randomized order. Reprinted from FEEST [23] under a CC BY license, with permission from Paul Ekman Group, LLC, original copyright 2002.

In the fMRI paradigm an event related design, optimised for maximal blood oxygen level dependent (BOLD) signal amplitude was used [24]. Basic emotions of anger, disgust, fear, sadness, surprise and happiness were presented. In total 24 stimuli of each of the six emotions (including emotion intensities of 50%, 75% and 100%, 8 stimuli of each), 45 neutral faces and 45 “meaningless” stimuli with random scattered patterns were presented in a randomized order. The stimuli were presented in three trials consisting of 26 stimulation sequences. Each stimulation sequence was 14s of duration consisting stimulus of epochs of 5s (a block of three stimuli of about 1s interleaved by rest epochs of about 1s) and followed by a rest period of 9s, similar to the methods described earlier [5] (Fig 1). Stimuli were presented via video goggles in pseudo-randomised order balanced with respect to the categories of basic emotions.

#### fMRI Data Acquisition and analysis

Images were acquired using a 3 Tesla whole body scanner (Symphony, Siemens, Erlangen, Germany). T1-weighted anatomical images and functional images were collected as described earlier [5]. To optimize data acquisition, imaging slice orientation was tilted by 30° [25].

Image processing was performed using SPM8 (Statistical parametric mapping, Wellcome Department of Imaging Neuroscience, London, UK) [26] as described previously [5]. The parameter estimates were modelled with six regressors for the basic emotions anger, disgust, fear, sadness, surprise and happiness. Each regressor was parametrically described according to intensity of emotional expression ranging from neutral to 50%, 75% and 100% (Fig 1). Regressors were convolved with a theoretical hemodynamic response function (hrf; sum of two gamma functions) [27]. The voxel time series were high pass filtered (time constant 141s) and the noise component in the model was described by a first order autoregressive model.

### Statistical analysis

All statistical analyses were performed with Statistical package for Social Sciences (SPSS version 21.0 IBM). Mean values ± standard deviations are given in the tables. One-way ANOVAs with between factor group (ALS patients, healthy controls) and within factors demographics, psychological adjustment (depression), and neuropsychological test performance were conducted. For behavioral performance of face recognition ability, ANCOVA with between factor group (ALS patients, healthy controls) and within subject factor facial emotion (percentage of correctly identified emotions) corrected for depressiveness was used. A threshold of p<0.05 was adopted for statistical significance.

For fMRI data, individual weighting factors of the emotion regressors for each participant and trial were computed. Individual parametric maps were subjected to a second level group (ALS patients vs. healthy controls) analysis using a two way ANOVA with emotional facial expressions for between-group differences.

The association between BOLD response and level of degree of social contacts was tested using a second level analysis of simple regression. Only areas with a significance of uncorrected p<0.005 at voxel level and with an extended cluster threshold ≥ 12 voxels were considered significant.

## Results

### Demographic, clinical, social and neuropsychological variables

ALS patients and healthy controls were matched with respect to demographics (Tables 1 and 2). ALS patients of the behavioural task presented with increased depression (BDI: p<0.01) compared to healthy controls (Table 1). The patients of the fMRI paradigm presented with decreased premorbid intelligence (p<0.01) and increased depression (BDI: p<0.01), compared to the healthy controls. No statistically significant difference was seen between patients and healthy controls in other neuropsychological variables or in degree of social contacts (Table 2).

**Table 1:** Participants of the behavioral emotion recognition task.

	ALS-patients N = 30	Healthy controls N = 29		
	mean±SD	mean±SD	F	p
Age [years]	60±10	61±8	0.39	0.54
Gender	16 females	8 females	4.2	0.05
Education [years]	9.7±1.5	10.0±1.7	0.83	0.37
Symptom onset	21 spinal	N/A		
ALS-FRS-R	27.9±11.4	N/A		
Disease duration [months]	41±18	N/A		
Progression rate	0.9 ± 0.7	N/A		
**Depression**				
BDI	12.9±4.9	6.2±4.0	32.24	<0.01*
**Dementia**				
MMSE	29.5±0.8	29.7±0.7	0.90	0.35

ALS-FRS-R: ALS functional rating scale revised [14]; BDI: Beck´s Depression Inventory; MMSE: Mini Mental State Examination.

* indicates statistical significance with p<0.05 in a two-sample t-test.

**Table 2:** Participants of the fMRI paradigm.

	ALS-patients N = 15	Healthy controls N = 14		
	mean±SD	mean±SD	F	p
Age [years]	54±12	61±10	2.9	0.10
Gender	5 females	7 females	0.79	0.38
Education [years]	10.1±1.6	10.8±1.6	0.83	0.37
Symptom onset	15 spinal	N/A		
ALS-FRS-R	28.7±9.6	N/A		
Disease duration [months]	33 ± 18	N/A		
Progression rate	1.0 ± 0.7	N/A		
Handedness	All right handed	All right handed		
**Depression**				
BDI	14.3±5.3	4.5±3.9	16.8	<0.01*
**Social contacts**				
Number	16±15	25±34	0.7	0.40
Hours	12.5±8.4	8.4±7.4	1.9	0.20
**Dementia**				
MMSE	29.6±0.7	29.1±0.9	2.3	0.14
**Premorbid crystallized verbal intelligence**				
MWT-B	98.0±12.6	117.4± 0.0	16.1	<0.01*
**Phonematic verbal fluency**				
single initial letter (“p”)	10.2±5.5	21.0±2.8	6.4	0.05
alternating initial letters (”g” and “r”)	15.2±4.9	17.0±4.2	0.2	0.67
**Semantic verbal fluency**				
animals	27.0±8.3	35.5±0.7	1.9	0.23
**Design fluency**				
5-point fluency	25.0±7.2	38.5±10.6	3.6	0.13
**Attention**				
SDMT	39.2±7.2	54.5±9.2	5.7	0.06

ALS-FRS-R: ALS functional rating scale revised version; BDI: Beck´s Depression Inventory; Number: people seen (at least for a one minute per person) on average per day; Hours: hours spent with people on average per day; MMSE: Mini Mental State Examination; MWT-B: Mehrfachwahl-Wortschatz-Intelligenztest-B, premorbid crystallized verbal intelligence test; Phonematic verbal fluency single initial letter (“p”): listing words with initial letter “p”, age-scaled percentile; verbal fluency alternating initial letters (“g” and “r”): listing alternating words with initial letters “g” and “r”, age-scaled percentile; Semantic verbal fluency animals: listing animals, age-scaled percentile; 5-Point fluency: non-verbal design fluency; SDMT: Symbol Digit Modalities Test—correct items.

* indicates statistical significance with p<0.05 in a two-sample t-test.

### Facial emotion processing at behavioural level

**Fig 2:**
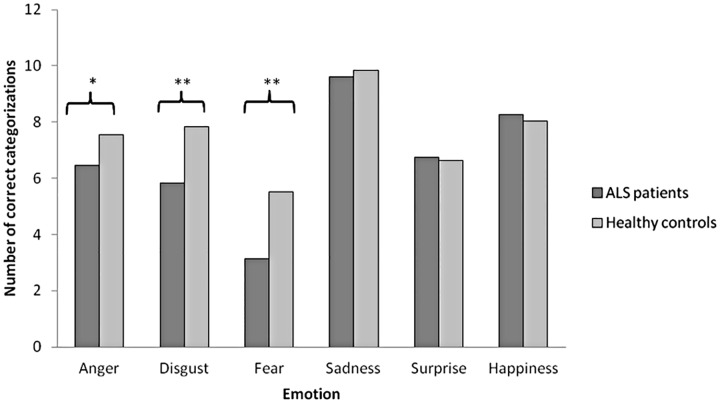
Number of correct categorizations of facial emotion expressions. * indicates statistical significance with p<0.05 and ** indicates statistical significance with p<0.01 between patients (n = 30) and healthy controls (n = 29) in a two-sample t-test.

ALS patients (n = 30) recognized anger (p = 0.04), disgust (p<0.01) and fear (p<0.01) less accurately than healthy controls (Fig 2, Table 3). Patient subgroup (n = 15) measured in fMRI recognized fear (p<0.01), disgust (p = 0.02) and happiness (p = 0.03) less than healthy controls (Table 3). However, analysis of covariance revealed that variance between the patients and the control groups in rating angry faces and happy faces were explained by the higher depression of the patients compared to healthy controls (p>0.05, when corrected for depression). Higher depression score of the patients did not explain the variance in rating of disgust and fear (both p<0.05 when corrected for depression).

**Table 3:** Means and standard deviations of correct categorization of facial stimuli in ALS patients and healthy controls of the emotion recognition task at behavioral level of all patients and patients that participated in fMRI.

	ALS patients N = 30	ALS patients (fMRI) N = 15	Healthy controls N = 29	ALS patients (n = 30) vs. healthy controls		ALS patients (n = 15) vs. healthy controls	
*Emotion*	*mean ± SD*	*mean ± SD*	*mean ± SD*	*F*	*p*	*F*	*p*
Anger	6.53 ± 2.68	6.80 ± 2.54	7.55 ± 2.13	4.6	0.04*	1.1	0.31
Disgust	6.27 ± 2.55	5.73 ± 3.03	7.83 ± 2.33	9.8	<0.01**	3.2	0.02*
Fear	3.23 ± 1.87	3.47 ± 2.07	5.52 ± 2.21	34.2	<0.01**	0.1	<0.01**
Happiness	9.63 ±0.67	9.40 ± 0.83	9.83 ± 0.47	2.5	0.11	6.9	0.03*
Sadness	6.70 ± 2.00	6.53 ± 2.23	6.62 ± 2.09	0.3	0.60	0.3	0.90
Surprise	8.23 ± 1.52	7.53 ± 1.73	8.03 ± 1.48	0.7	0.39	0.3	0.32

* indicates statistical significance with p<0.05

** with p<0.01 in a two-sample t-test.

### BOLD-Response to emotional facial expressions: ALS patients versus controls

**Fig 3:**
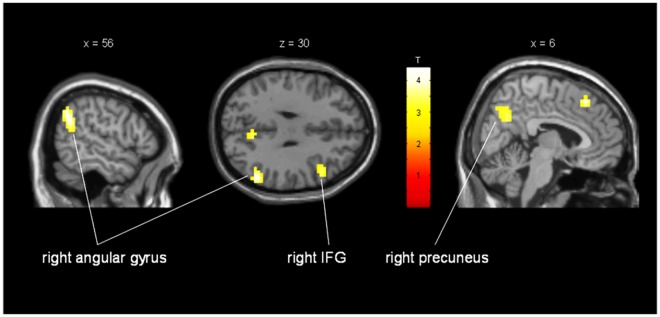
Increased activation of ALS patients compared to healthy controls when processing different emotional facial expressions. Activation in a sub region of the right inferior frontal gyrus (MNI coordinates: x = 49mm y = 18mm z = 30mm; cluster size = 37 voxels; T = 3.53; p_uncorr_ = 0.001), right angular gyrus (MNI coordinates: x = 56mm y = -58mm z = 34mm; cluster size = 102 voxels; T = 4.40; p_uncorr_<0.001) and right precuneus (MNI coordinates: x = 6mm y = -62mm z = 34mm; cluster size = 31 voxels; T = 3.42; p_uncorr_ = 0.001). Activations show areas with significant increase of activity in patients in average for all emotions.

During the fMRI task of processing all types of emotional facial expressions, ALS patients presented with a significantly increased activity in right inferior frontal gyrus (BA 44/45). In addition, patients showed increased activity in right angular gyrus, right insula and right precuneus (Fig 3; Table 4). In comparison, the patients presented with significantly decreased activity in inferior frontal gyrus, orbitofrontal gyrus, precentral gyrus, middle temporal gyrus and calcarine sulcus on the left side and in lingual gyrus, sub-lobar frontal gyrus, and cerebellum on the right side (Table 5).

**Table 4:** Regions of increased activation in processing emotional facial stimuli in ALS patients compared to healthy controls (all emotions averaged).

Area	Left/Right	MNI Coordinates	Cluster Size	T	p_uncorr_
Angular Gyrus	R	56–58 34	102	4.40	<0.001
Insula	R	38 28 5	16	3.77	<0.001
IFG	R	49 18 30	37	3.53	0.001
Precuneus	R	6–62 34	31	3.42	0.001

Displayed are clusters >15 voxels with uncorrected threshold of p<0.001; IFG = Inferior Frontal Gyrus

**Table 5:** Regions of decreased activation in processing emotional facial stimuli in ALS patients compared to healthy controls (all emotions averaged).

Area	Left/Right	MNI Coordinates	Cluster Size	T	p_uncorr_
OFG	L	-26 39–16	98	7.47	<0.001
MTG	L	-55–54 0	283	6.25	<0.001
		-59–40 9	28	4.92	<0.001
Precentral Gyrus	L	-44 7 51	42	5.48	<0.001
Lingual Gyrus	R	13–87–8	44	5.17	<0.001
IFG	L	-44 36 26	70	5.07	<0.001
sub-lobar Frontal Gyrus	R	17 32–8	20	4.94	<0.001
Calcarine Sulcus	L	-26–58–9	43	4.61	<0.001
Cerebellum	R	31–54–42	25	4.56	<0.001

Displayed are clusters >15 voxels with uncorrected threshold of p<0.001; IFG = Inferior Frontal Gyrus; OFG = Orbitofrontal Gyrus; MTG = Middle Temporal Gyrus

#### Response to different types of emotional facial expressions: ALS patients versus controls

Significant differences between the ALS patients and healthy controls were seen only for sad faces. Compared to the healthy controls ALS patients presented with increased activity in the right inferior frontal gyrus (p<0.005) but decreased activity in hippocampus bilaterally (p<0.005), when processing images of sad faces. No significant differences in brain activity between the patients and healthy controls were seen for any of the other emotional expressions.

#### Brain responses to emotional facial expressions: association with cognition and social contacts

Premorbid intelligence was the only assessed neuropsychological domain where the patients presented with significantly lower scores than the controls. However, a post-hoc regression analysis of fMRI responses and premorbid intelligence provided no association of premorbid intelligence and the number of activated voxels in the patients’ brain areas with decreased or increased activation.

**Fig 4:**
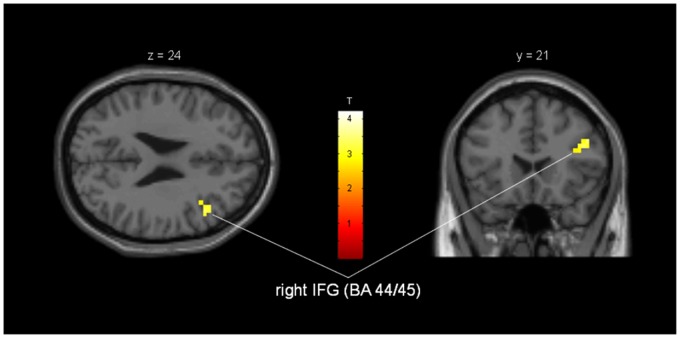
Regression analysis of brain activity and social contacts during processing of emotional facial stimuli in ALS patients. Activation in a subregion of right inferior frontal gyrus/Brodmann Area 44 and 45. MNI-coordinates: x = 44mm, y = 21mm, z = 30mm; cluster-size = 12 voxels; T = 3.36; p_uncorr_ = 0.003.

Regression analysis revealed a statistically significant positive correlation between number of social contacts and activity in right inferior frontal gyrus (BA 44/45; p = 0.003) for ALS patients (Fig 4). This correlation was not seen for healthy controls.

## Discussion

In the current study, faces of the six basic emotions were presented to medium and advanced affected ALS patients and healthy controls. Data of correct emotion categorization and cortical BOLD responses in fMRI were recorded.

Patients showed reduced performance in facial emotion recognition for disgust and fear. Patients presented with increased activity in the right inferior frontal gyrus and decreased activity in hippocampus bilaterally during processing of emotions in faces, especially for sadness. Activity in the right inferior frontal gyrus was positively correlated with number of daily social contacts.

ALS patients reduced recognition of disgust and fear might be interpreted as a change in the network of facial emotion processing. Lower cognitive abilities of ALS patients suggested earlier [28] most likely do not account for the changes in emotion recognition, as most of the tests assessing cognition did not reveal significant difference between the patients and the controls.

During the fMRI task of processing facial expressions, patients showed decreased activity in areas related to the emotions they also recognized less. Reduced brain activity was seen i.e. in left middle temporal and left precentral gyrus that might be involved in processing of negative facial expressions like disgust [29]. Furthermore, patients showed reduced activity in left inferior frontal gyrus (BA 44/45), an area known to be involved in processing of facial expressions of anger and fear [30].

When analyzing brain activities for each emotion separately, differences between the patients and controls were found only for sad faces. When processing sad faces patients showed increased brain activity in right inferior frontal gyrus (BA 44/45), an area associated with imitating emotional responses, also often called “mirror neuron area” [31] and decreased activity in hippocampus bilaterally.

Hippocampus is a main target for storage and retrieval of information and memory [32] and it might be affected in the course of ALS [1] thus being associated with memory impairment in ALS [33]. Healthy controls might use hippocampus for memory retrieval to correctly categorize emotional expressions [34], whereas patients may have impaired access to this retrieval loop. Alternatively, the patients might increase the activation in the right inferior frontal gyrus, which is also known to be important for facial expression recognition [35].

However, this all remains highly speculative as hippocampus is considered to be mainly involved in retrieval of recent memories [36] which is not the case in facial emotion recognition. Also the patients of the current study did not show any memory deficits. Alternatively, other aspects might account for the change in cortical activity. Several studies have reported involvement of cortical hyper excitability [37,38,39] and reduced corticocortical inhibition [40] in ALS. It has also been suggested that not just hyper excitability but imbalance between cortical excitation and inhibition might take place in ALS [41]. Most likely these pathological changes also increase in the course of the disease being more prominent in advanced ALS, which might partly explain brain activity differences between patients and healthy controls in the current study.

Some have suggested cortical reorganisation in different cerebral networks in ALS [1,12]. Functional cortical connectivity may be reduced [42] or alternatively increased [43] in some brain areas of ALS patients. Until now, there has been no evidence for a functional relevance of these reorganisation processes. Therefore, this is to our knowledge the first study on emotional processing in ALS suggesting that in the course of ALS increased activity (in right inferior frontal areas) might be considered as functional compensation and reorganization in the best sense.

Furthermore, the increased activity in inferior frontal gyrus was associated with increased number of daily social contacts of ALS patients. This suggests that positive impact of social contacts on affective state might be reflected in the inferior frontal gyrus network activity. It has been suggested that social contacts are a protective factor against cognitive decline [44]. Thus, neurodegenerative processes in the course of ALS might be counteracted by positive emotional activity in social life, possibly via the indirect pathway of reducing depression in patients at later stages of the disease.

The ALS sample investigated here showed increased depression compared to the healthy sample. Depressive mood increases after the diagnosis but even already one year before the diagnosis patients may show increased depression [45]. However, often in the course of the disease acceptance of artificial respiration, quality of life and depression draw closer to the level of the healthy population [15,46,47]. In the later phases of the disease patient’s attention is focused on caretaking family members [15] and many caretakers show a positive attitude and positive emotional responsiveness to the patient thus increasing the force of the positive emotional-social buffer [48].

These data replicate our earlier results with a comparable group of ALS patients using the IAPS. We found increased positive affective responding to positive slides and decreased negative affective responding to negative emotional slides in ALS compared to the matched controls [4]. In another study of ours, ALS patients demonstrated positive subjective responding and increased activity in the supramarginal gyrus in the course of the disease [9]. The supramarginal gyrus just as the right inferior frontal gyrus can be considered as part of the brain network related to positive emotional-social perception [9]. Overall, the current study provides intriguing evidence for the importance of including ALS patients in social life to counteract possible negative effects of pathological changes on social-emotional information processing.

## Limitations

A shortcoming of our study is the limited number of patients. However, the criterion of a homogenous group and the time consuming investigations were limiting factors for participation. Furthermore, patients showed reduced scores in premorbid crystallized verbal intelligence, which however was unlikely affecting the performance of the patients as we found no correlation of premorbid intelligence and the number of activated voxels in the areas described. Patients were also able to perform the task properly and presented with increased activity in prefrontal areas rather than reduced activity as it might be expected in case of cognitive deficits or FTD.

Additionally, patient cohort presented with increased depression compared to controls. It has been shown that depressed individuals are more reactive to sad faces [49] and might direct more attention to images expressing sadness [50] which could explain increased cortical activation when viewing sad faces. Furthermore, depression is associated with impaired memory [51] and reduced hippocampal volume [52] which might lead to reduced hippocampal activation as seen in the patients of the current study. Additionally, depressed individuals might have difficulties with selective attention [50], which may reduce performance in the emotion recognition task. However, in the current sample there was no evidence for globally impaired attention performance, neither in neuropsychological assessment nor in the facial recognition task.

Furthermore, unlike mostly in the studies on depression none of our patients presented with clinically relevant depression according to the criteria of the Diagnostic and Statistical Manual of Mental Disorders (DSM-5) [53]. Despite the significant difference between the patients and healthy controls in depression score, the patient cohort presented only with mild depression, some having no depressive symptoms at all. Therefore, it is unlikely that patients’ increased negative mood heavily influenced their emotion processing at behavioural or cortical level.

## Supporting Information

S1 File Copyright permission for Fig 1. (PDF)

S2 File Questionnaire on social contacts in English. (DOCX)

S3 File Questionnaire on social contacts in German. (DOCX)
