# Supplementary material for: Perception of Emotional Facial Expressions in Amyotrophic Lateral Sclerosis (ALS) at Behavioural and Brain Metabolic Level
Source: PLoS One. 2016 Oct 14;11(10):e0164655. doi: 10.1371/journal.pone.0164655 (PMC5065224; doi:10.1371/journal.pone.0164655)
Supplement: S1 File — (PDF) [file pone.0164655.s001.pdf]

Paul Ekman Group, LLC.

PO Box 26089
San Francisco, CA, 94126-6089
EIN: 20-1170753

Invoice #: 2850

Date: August 2, 2016

Bill To/Contact:

PD Dr. Dorothée Lulé,
University of Ulm, RKU,
Department of Neurology
Oberer Eselsberg 45
89081 Ulm
Germany

Phone: 0049 (0) 731 177 5267

dorothee.lule@uni-ulm.de

Publisher: PLOSONE
Book/publication title: PLOSONE
Article title: Perception of emotional facial expressions in
amyotrophic lateral sclerosis (ALS) at behavioural and brain metabolic level
Book/article/publication author(s): Aho-Özhan,Keller, Heimrath,Uttner, Kassubek, Birbaumer,Ludolph, Lulé
First edition or reprint: first edition
Anticipated publication date: 22.08.2016
Anticipated print run/number of copies: 10
Format (print and/or electronic): electronic

Quantity	Item Code	Description	Cost
1	POFA	Pictures of Facial Affect	$175
		**Total**	**$175**

Preferred **- Pay By Credit Card:**

Contact us at custserv@paulekman.com to get instructions for paying on our secure website: www.paulekman.com

Pay by Check:

--Payable to Paul Ekman Group

--Please ensure that our invoice number is on the check or stub

--PO Box Address, ATTN: Permissions Dept.

THIS INVOICE ENTITLES THE ABOVE PERSON/ORGANIZATION PERMISSION TO USE THE IMAGE(S) IN THE PUBLICATION AS STATED ABOVE FOR THE STATED EDITION ONLY. SUBSEQUENT EDITIONS REQUIRE ADDITIONAL PERMISSION/INVOICING. ALL RIGHTS GRANTED ARE NON-EXCLUSIVE. PAYER AGREES TO ENSURE PROPER ACCREDITATION TO: PAUL EKMAN, PH.D./ PAUL EKMAN GROUP, LLC.
PLEASE CONTACT US AT CUSTSERV@PAULEKMAN.COM FOR FURTHER DETAILS.
