# Supplementary material for: Perception of Emotional Facial Expressions in Amyotrophic Lateral Sclerosis (ALS) at Behavioural and Brain Metabolic Level
Source: PLoS One. 2016 Oct 14;11(10):e0164655. doi: 10.1371/journal.pone.0164655 (PMC5065224; doi:10.1371/journal.pone.0164655)
Supplement: S2 File — (DOCX) [file pone.0164655.s002.docx]

**General questions about social contacts**

1. Estimate the average number of people you see daily.
2. Estimate the number of hours that you spend daily on average with other people (possibly with some more than one hour).
3. Are you in a relationship? If yes, do you live together?
4. Are you employed?
5. Do you have children? If yes, do they live with you?
6. Are you a member of any association? If yes, of how many?
