# Supplementary material for: Perception of Emotional Facial Expressions in Amyotrophic Lateral Sclerosis (ALS) at Behavioural and Brain Metabolic Level
Source: PLoS One. 2016 Oct 14;11(10):e0164655. doi: 10.1371/journal.pone.0164655 (PMC5065224; doi:10.1371/journal.pone.0164655)
Supplement: S3 File — (DOCX) [file pone.0164655.s003.docx]

**Allgemeine Fragen zu den sozialen Kontakten**

1. Schätzen Sie die Anzahl der Menschen ein, die Sie täglich im Durchschnitt sehen.
2. Schätzen Sie die Anzahl der Stunden ein, die Sie täglich im Durchschnitt Kontakte mit Menschen haben (ein Mensch evtl. mehrere Stunden).
3. Leben Sie in einer festen Partnerschaft? Wohnen Sie in einer gemeinsamen Wohnung?
4. Sind Sie berufstätig?
5. Haben Sie Kinder? Leben diese bei Ihnen?
6. Sind Sie Mitglied in einem Verein? In wie vielen?
